# Systemic lupus erythematosus and prostate cancer risk: a pool of cohort studies and Mendelian randomization analysis

**DOI:** 10.1007/s00432-023-04853-5

**Published:** 2023-05-22

**Authors:** Junyong Ou, Kailan Zhen, Yaqian Wu, Zixuan Xue, Yangyi Fang, Qiming Zhang, Hai Bi, Xiaojun Tian, Lulin Ma, Cheng Liu

**Affiliations:** 1 Department of Urology, Peking University Third Hospital, Peking University Health Science Center, 49 North Garden Road, Beijing, 100191 China; 2 Department of Histology and Embryology, Southern Medical University, Guangzhou, 510515 China; 3 Department of Urology, Shanghai General Hospital, Shanghai Jiao Tong University School of Medicine, 85 Wujin Road, Shanghai, 200080 China

**Keywords:** Prostate cancer, Systemic lupus erythematosus, Risk, Genetic variants, Genome-wide association studies, Mendelian randomization

## Abstract

**Background:**

Current observational studies suggest that there may be a causal relationship between systemic lupus erythematosus (SLE) and prostate cancer (PC). However, there is contradictory evidence. This study aimed to investigate and clarify the association between SLE and PC.

**Methods:**

We searched PubMed, Embase, Web of Science, and Scopus until May 2022. A meta-analysis was conducted on the standard incidence rate (SIR) and 95% CI. Subgroup analysis was performed based on the follow-up duration, study quality, and appropriate SLE diagnosis. Mendelian randomization (MR) of the two samples was used to determine whether genetically elevated SLE was causal for PC. Summary MR data were obtained from published GWASs, which included 1,959,032 individuals. The results were subjected to sensitivity analysis to verify their reliability.

**Results:**

In a meta-analysis of 79,316 participants from 14 trials, we discovered that patients with SLE had decreased PC risk (SIR, 0.78; 95% CI, 0.70–0.87) significantly. The MR results showed that a one-SD increase in genetic susceptibility to SLE significantly reduced PC risk (OR, 0.9829; 95% CI, 0.9715–0.9943; *P* = 0.003). Additional MR analyses suggested that the use of immunosuppressants (ISs) (OR, 1.1073; 95% CI, 1.0538–1.1634; *P* < 0.001), but not glucocorticoids (GCs) or non-steroidal anti-inflammatory drugs (NSAIDs), which were associated with increased PC risk. The results of the sensitivity analyses were stable, and there was no evidence of directional pleiotropy.

**Conclusions:**

Our results suggest that patients with SLE have a lower risk of developing PC. Additional MR analyses indicated that genetic susceptibility to the use of ISs, but not GCs or NSAIDs, was associated with increased PC risk. This finding enriches our understanding of the potential risk factors for PC in patients with SLE. Further study is required to reach more definitive conclusions regarding these mechanisms.

**Supplementary Information:**

The online version contains supplementary material available at 10.1007/s00432-023-04853-5.

## Introduction

Systemic lupus erythematosus (SLE) is a chronic autoimmune inflammatory disease that is characterized by the generation of autoantibodies, complement activation, and complex immune deposits, resulting in almost complete tissue and organ damage (Fava and Petri 2019). SLE mainly occurs in women, with a female-to-male ratio of 10:1 (Yang et al. 2019), and is most prevalent in people of North American and African ethnicity (Nusbaum et al. 2020). As sex hormone levels decrease in men with SLE, SLE-induced changes in the androgen pathway may reduce prostate cancer (PC) risk. Therefore, SLE may be associated with morbidity and mortality from PC (Mok and Lau 2000).

PC is the second most frequent malignancy and the sixth major cause of cancer-related fatalities in men globally. In 2018, there were an estimated 1.276 million new patients and 359,000 deaths from PC, and these figures are expected to grow to approximately 2.3 million new cases and 740,000 fatalities by 2040 (Bray et al. 2018). Therefore, early detection of suspected at-risk patients and timely disease intervention are critical for reducing PC incidence and mortality (Barry and Simmons 2017).

To date, many studies have attempted to evaluate overall PC risk in patients with SLE. However, these results are neither comprehensive nor consistent (Bao et al. 2014; Cao et al. 2015; Mao et al. 2016; Yeo et al. 2020). SLE predominantly occurs in women (Mellemkjér et al. 1997), whereas PC is common in men aged ≥ 65 years (Patel and Klein 2009); therefore, there may not be sufficient data to study PC in patients with SLE. The observational studies cannot infer causality from the association between SLE and PC, as this may be affected by reverse causality or confounding factors (high body mass index, alcohol consumption, smoking, and vitamin D supplementation) (Pernar et al. 2018). SLE treatment relies heavily on the use of non-steroidal anti-inflammatory drugs (NSAIDs), immunosuppressants (ISs), and glucocorticoids (GCs) (Fava and Petri 2019), which may act as confounding factors in PC development. Thus, it is still difficult to draw a definitive conclusion.

Considering the shortcomings of observational studies and the relatively long developmental duration between SLE and PC, investigating causal relationships through randomized controlled trials (RCTs) is a logical next step. The decomposition of Mendelian randomization (MR) is a new epidemiological method that can provide an analog to RCTs (Smith and Ebrahim 2004). Furthermore, through Mendel's second law, MR can eliminate the influence of confounding factors using single-nucleotide polymorphisms (SNPs) as instrumental variables (IVs). Therefore, we performed an updated meta-analysis and MR analysis to evaluate the possible causal relationship between SLE and PC risk.

## Methods

### Meta-analysis

Before this meta-analysis, a study protocol (CRD42022336182) was announced on the Prospero website. The meta-analysis was performed following published guidelines for meta-analyses of observational studies in epidemiology (MOOSE) (Stroup et al. 2000).

#### Literature search

Related articles were searched on PubMed, Embase, Web of Science, Scopus, and Cochrane libraries until May 2022. Two researchers used a combination of MESH search terms for retrieval: ‘‘systemic lupus erythematosus, cancer, risk, incidence, cohort,” and entry. Only studies published in English were included in this meta-analysis.

#### Eligibility criteria

The inclusion criteria were as follows: (1) observational or cohort studies; (2) studies providing a standardized incidence rate (SIR), odds ratio (OR), hazard ratio (HR), or relative risk (RR) with a corresponding CI of PC incidence in patients with SLE; and (3) studies with eligible follow-up times (four years). The exclusion criteria were as follows: (1) reviews, case reports, letters, or expert opinions; and (2) studies that did not provide data on PC or SLE.

#### Data acquisition and quality evaluation

Two qualified researchers, Jun-Yong Ou and Kai-Lan Zhen, read the full texts and evaluated the quality of each study. Disagreements were settled through discussion. We extracted data, such as the author, year, sources, follow-up duration, diagnosis, number of patients with SLE (men and women), and SIR with a 95% CI. The quality of each study was evaluated by the quality assessment tool for systematic reviews of observational studies (QATSO) (Wong et al. 2008). The scoring system ranged from 0 to 6, with 0 and 6 being the lowest and highest quality, respectively. In each investigation, six areas were evaluated as follows: (1) eligibility criteria for selecting participants; (2) appropriate SLE diagnosis; (3) participant characteristics; (4) ascertainment of PC; (5) adjustments for age and sex; and (6) other relevant adjustments.

#### Statistical analysis.

Given that the prostate cancer risk is relatively low among patients with SLE, the authors anticipated similar SIR estimates with HRs/ORs/RRs, as described by Lin et al*.* (2018). The meta-anathrough SLE, we excluded genetic associationlysis collected study-specific ORs/HRs/RRs/SIRs and converted them into SIRs with corresponding 95% CI for PC in order to combine the data. The SIR was calculated as the ratio of the observed to the expected number of cancers. The expected numbers of cancers for men and women were calculated separately. The expected number of malignancies was calculated as follows:

$${\text{E }} = {\text{ S }}\left( {{\text{ni}}} \right) \, \times {\text{ Ri,}}$$

where E is the expected number of malignancies, S (ni) is the sum of all person-years at risk in age group i from the study cohort, and Ri is the age- and sex-specific cancer rates for cohort districts in age group i. A fixed-effects model was used if there was no significant heterogeneity (*P* > 0.5, I^2^ < 50%); otherwise, a random-effects model was used. The findings of multiple similar trials were heterogeneous when the p-value of the Q statistic was 0.10. I^2^ values of 25%, 50%, and 75% indicated low, medium, and high degrees of heterogeneity, respectively. Publication bias was investigated using funnel plots and Begg's test. Additionally, we performed meta-regression to determine the sources of heterogeneity. Sensitivity analysis was performed by the sequential removal of each study. We considered that different follow-up durations, study qualities, and appropriate SLE diagnoses might have affected the conclusions; subgroup analyses were performed based on these factors. Statistical significance was set at *P* < *0.05*. Meta-analysis was performed using STATA software (version 17.0).

### Mendelian randomization

**Fig. 1:**
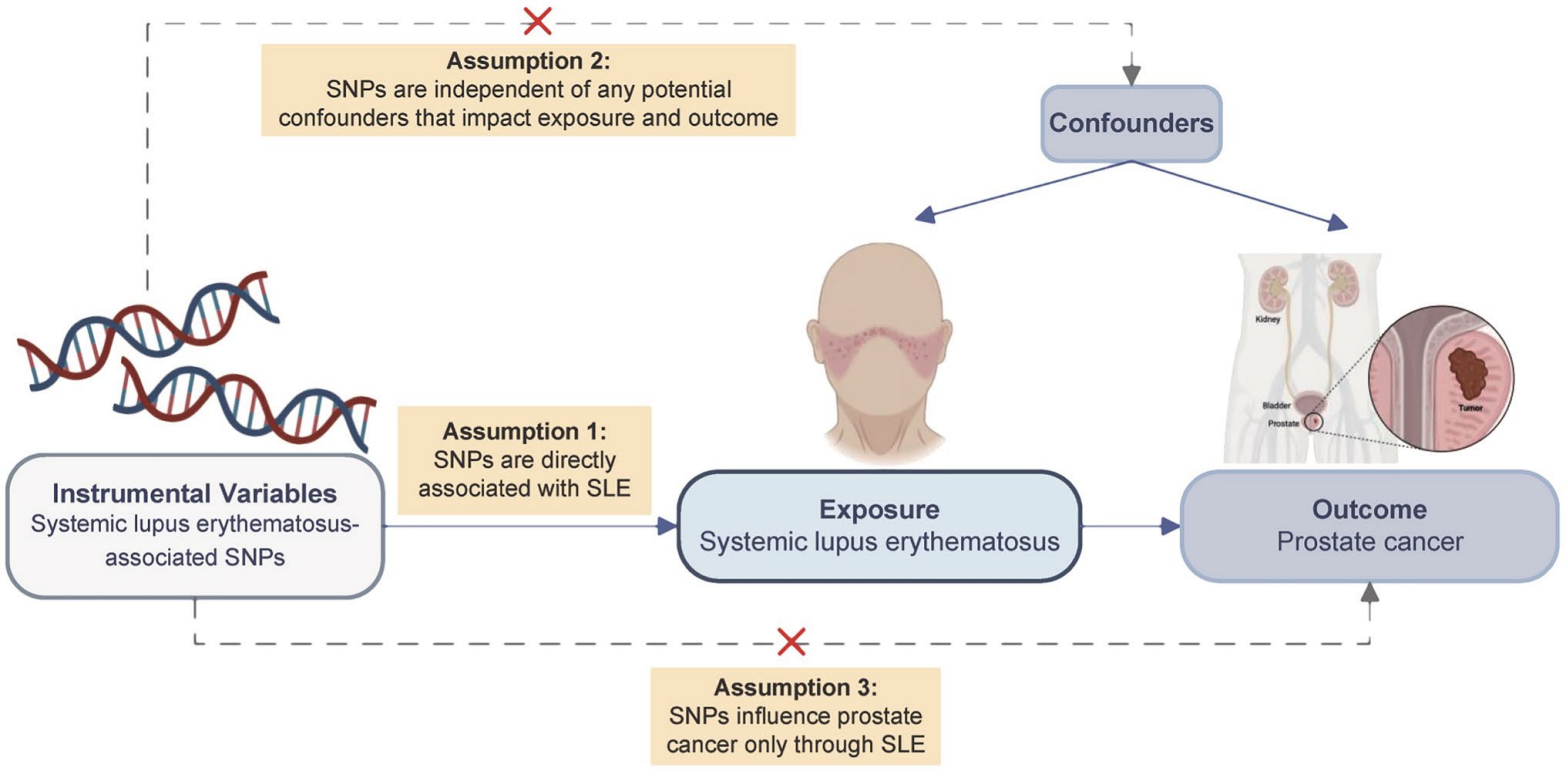
Illustrative diagram of Mendelian randomization assumptions. SNP, single-nucleotide polymorphism; SLE, systemic lupus erythematosus

The MR guidelines followed in our study are based on three basic assumptions: (1) genetic markers are closely related to SLE; (2) IVs are independent of confounding factors between SLE and PC; and (3) IVs influence PC only through their effects on SLE and not through other alternative causal pathways. Therefore, genetic markers are not pleiotropic through pathways other than exposure. None of these assumptions could be violated; otherwise, the causal links drawn from MR studies would not have been sufficiently reliable. A summary of the MR study design is shown in Fig. 1.

#### Genetic variants associated with SLE

Summary data of genetic variants associated with SLE were retrieved from published genome-wide association studies (GWASs), including 5,201 cases and 9,066 controls by Bentham et al (2015) (Table 1). All participants were of European ancestry. Forty-five genetic variants were determined to have genome-wide significance (*P* < 5E-8). To determine whether the SNPs were solely related to PC through SLE, we excluded genetic association confounders using the PhenoScannerV2 website (www.phenoscanner.medschl.ca.ac.uk). Genetic confounders associated with PC include high body mass index, alcohol consumption, smoking, vitamin D supplementation (Pernar et al. 2018), and medication use (GC, IS, and NSAIDs) (1). In addition, we utilized linkage disequilibrium (LD) analysis to exclude SNPs when mutual LD exceeded the limit (kb = 5000, R^2 ^<0.01). Finally, 48 SNPs were applied to the IV instruments (Table S1), which explained 6.45% of SLE variation. The F-statistic of 12,592.46 (> 10) indicated a strong prediction of the SLE instruments used.

**Table 1:** Characteristics of the included GWAS summary studies in Mendelian randomization

Trait	Pubmed ID	First author	Consortium	Sex/population	Sample size	Number of cases	Number of controls	Year	GWAS ID
Exposure									
Systemic lupus erythematosus	26,502,338	Bentham J	NA	NA/European	14,267	5201	9066	2015	ebi-a-GCST003156
Outcome									
Prostate cancer	29,892,016	Schumacher	PRACTICAL	Male/European	140,254	79,148	61,106	2018	ebi-a-GCST006085
Confounders									
Obesity class 1	23,563,607	Berndt SI	GIANT	Male and female/European	98,697	32,858	65,839	2013	ieu-a-90
Obesity class 2	23,563,607	Berndt SI	GIANT	Male and female/European	72,546	9889	62,657	2013	ieu-a-91
Obesity class 3	23,563,607	Berndt SI	GIANT	Male and female/European	50,364	2896	47,468	2013	ieu-a-92
Ever smoked	NA	Ben Elsworth	MRC-IEU	Male and female/European	461,066	280,508	180,558	2018	ukb-b-20261
Former alcohol drinker	NA	Ben Elsworth	MRC-IEU	Male and female/European	31,506	16,191	15,315	2018	ukb-b-12654
Vitamin D supplements	NA	Ben Elsworth	MRC-IEU	Male and female/European	460,351	17,879	442,472	2018	ukb-b-12648
Medication use (GCs)	31,015,401	Wu Y	UK Biobank	Male and female/European	205,700	17,352	188,348	2019	G9IYjw
Medication use (ISs)	31,015,401	Wu Y	UK Biobank	Male and female/European	272,602	3954	268,648	2019	qB4cjr
Medication use (NSAIDs)	31,015,401	Wu Y	UK Biobank	Male and female/European	164,520	74,150	90,370	2019	CEJibS

*GWASs* genome-wide association studies, *PRACTICAL* Prostate Cancer Association Group to Investigate Cancer Associated Alterations in the Genome, *GIANT* genetic investigation of anthropometric traits consortium, *MRC-IEU* MRC Integrative Epidemiology Unit, *GCs* glucocorticoids, *ISs* immunosuppressants, *NSAIDs* non-steroidal anti-inflammatory drugs, *NA* not available

#### GWAS summary data on PC

Summary statistics for PC in people with European ancestry were obtainedAll of the above findings suggest a robust estimate

from the Prostate Cancer Association Group to Investigate Cancer Associated Alterations in the Genome (PRACTICAL) Consortium (79,148 PC and 61,106 control cases) (Fr et al. 2018). Written consent was obtained from all participants. The appropriate ethical review boards supported all studies, and data were extracted for MR analysis. Table 1 lists the main features of the included GWASs.

#### Statistical analysis

The Wald-type inverse variance weighting (IVW) method for random effects estimates the influence of exposure on the outcomes. The results are expressed as an OR and 95% CI. Other MR approaches, such as MR-Egger, weighted median, and weighted mode, were applied to examine the consistency of effect estimation.

The first assumption was satisfied because we selected SNPs at a genome-wide significance threshold of *P* < 5E-8 and the F-statistic was 12,592.46 (> 10). To verify the second assumption, we employed additional MR methods to analyze the potential confounding factors that might affect SLE and PC progression. First, we retrieved genetic effects on obesity levels from the Genetic Investigation of Anthropometric Traits consortium (Berndt et al. 2013). Second, smoking, alcohol consumption, and vitamin D supplementation status were retrieved from the MRC-IEU for the entire UK Biobank (version March 3, 2018) genetic data (Bentham et al. 2015) (Table 1). Third, we employed several methods to test and control for horizontal pleiotropy. We conducted heterogeneity, potential horizontal pleiotropy, and leave-one-out tests for the sensitivity analysis. All MR analyses were performed using the two-sample MR package in R (version 4.1.0) (Hemani et al. 2018).

## Result

### Meta-analysis results

#### Study characteristics

**Fig. 2:**
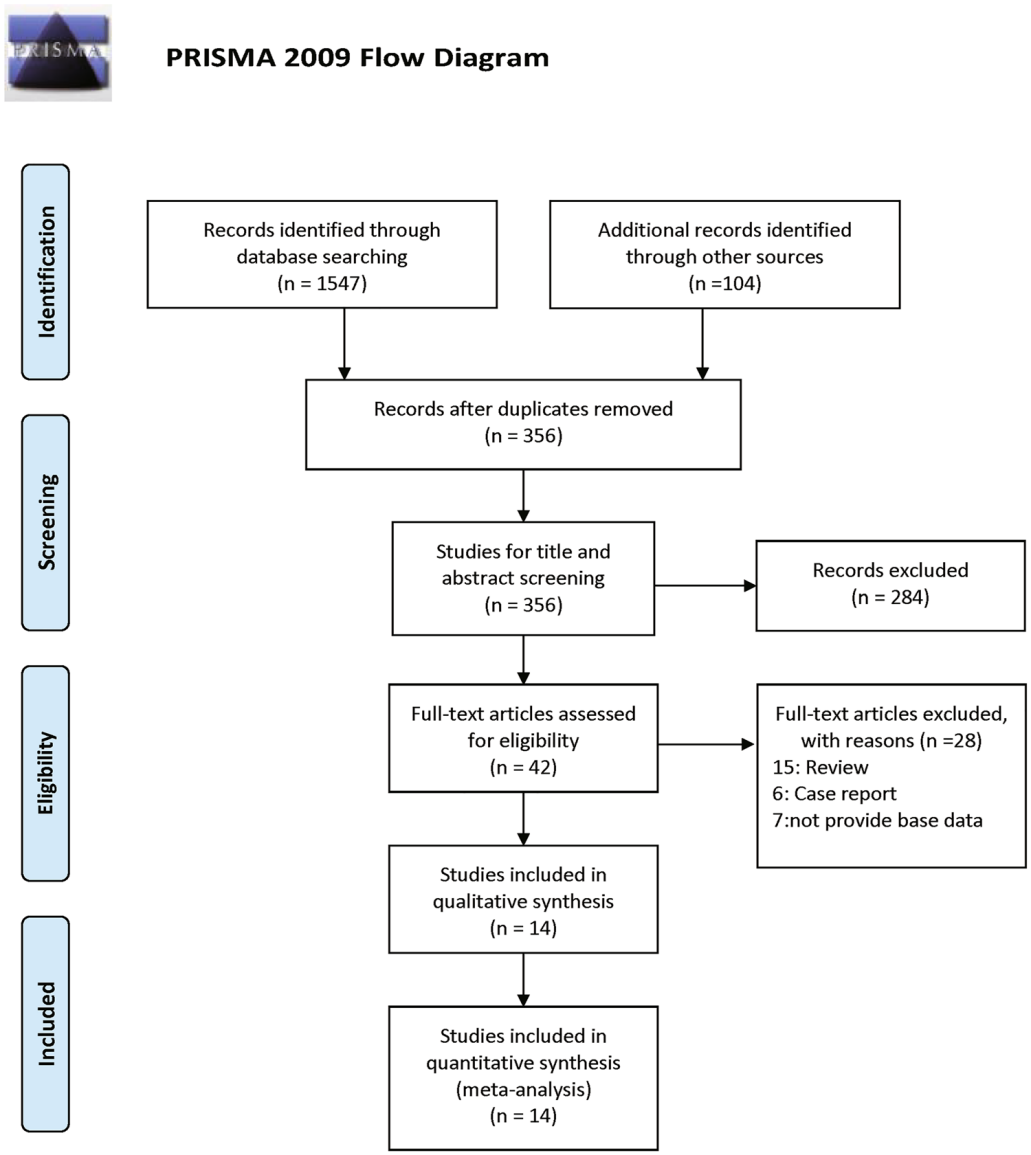
PRISMA 2009 flow diagram

A total of 1,651 related articles were obtained from the five databases. Of these, 356 were retained after deleting duplicate articles. Two hundred and eighty-four publications were eliminated because the title and abstract were irrelevant to the study content. A further 28 studies were excluded after reviewing the full text. Finally, 14 studies (Mellemkjér et al. 1997; Sultan et al. 2000; Nived et al. 2023; Cibere et al. 2001; Björnådal et al. 2002; Ragnarsson et al. 2003; Parikh-Patel et al. 2008; Chen et al. 2010; Dreyer et al. 2011; Bernatsky et al. 2013; Dey et al. 2013; Liu et al. 2013; Tallbacka et al. 2018; Westermann et al. 2021), including 79,316 patients with SLE published between 1997 and 2021, were considered eligible for inclusion in the study (Fig. 2). The mean and median follow-up times ranged from 4.8 to 25.7 years. Table 2 lists the basic features of these articles.

**Table 2:** Characteristics of the studies included in the meta-analysis

	Sources of patients with SLE	Mean or median follow-up (years)	SLE diagnosis	No. of SLE patients (male, female)	SIR (95% CI)
Mellemkjér et al. (1997)	Danish hospital discharge register	6.8	ACR criteria	1585 (269,1316)	0.59 (0.01–3.28)
(Sultan et al. 2000)	Board of health and welfare recorded data	4.8	ARA criteria	276 (18,258)	1.34 (0.03–7.46)
Nived et al. (2023)	SLE cohort registry	6.5	ACR criteria	116 (17,99)	2.86 (0.1–15.9)
Cibere et al. (2001)	University-based rheumatic disease unit	12.0	ACR criteria	294 (46,248)	1.81 (0.02–10.11)
Björnådal et al. (2002)	Swedish national board of health and welfare recorded data	8.8	NA	5715 (1514,4201)	0.77 (0.51–1.11)
Ragnarsson et al. (2003)	Icelandic SLE database	12.8	ARA criteria	238 (25,213)	1.22 (0.03,6.17)
Parikh-Patel et al. (2008)	California patient discharge data	5.1	NA	30,478 (3345,27,133)	0.69 (0.50,0.93)
Chen et al. (2010)	National health Insurance research database	6.1	ARA criteria	11,763 (1369,10,394)	0.79 (0.68–0.90)
Dreyer et al. (2011)	Danish central population register	13.2	ACR criteria	576 (68,508)	2.10 (0.30,15.00)
Bernatsky et al. (2013)	Multicenter clinical	7.4	ACR criteria	16,409 (1641,14,768)	0.65 (0.32–1.16)
Dey et al. (2013)	University college london hospitals lupus data	14.7	ACR criteria	595/NA	4.29 (1.09,10.24)
Liu et al. (2013)	Swedish hospital discharge registry	11.3	NA	7642/NA	1.03 (0.73,1.41)
Tallbacka et al. (2018)	Finland helsinki university central hospital	25.7	ARA criteria	205 (23,182)	0.40 (0.01,9.00)
Westermann et al. (2021)	Danish national patient register	8.1	NA	3424 (545,2879)	0.75 (0.32,1.48)

*SLE* systemic lupus erythematosus, *SIR* standard incidence rate, *ACR* American College of Rheumatology, *ARA* American Rheumatism Association *NA* not available *SLE* systemic lupus erythematosus, *SIR* standard incidence rate, *ACR* American College of Rheumatology, *ARA* American Rheumatism Association, *NA* not available

#### Quality evaluation

All quality scores ranged from four to six. Nine of the 14 studies (64%) scored > 5 points, indicating high quality. Two studies satisfied all the criteria for quality evaluation. Regarding SLE diagnosis, six studies (43%) used the American College of Rheumatology (ACR) criteria (Hochberg 1997), and four studies (29%) used the American Rheumatism Association (ARA) criteria (Alarcón-Segovia et al. 1992) (Table S1).

#### PC risk in SLE

**Fig. 3:**
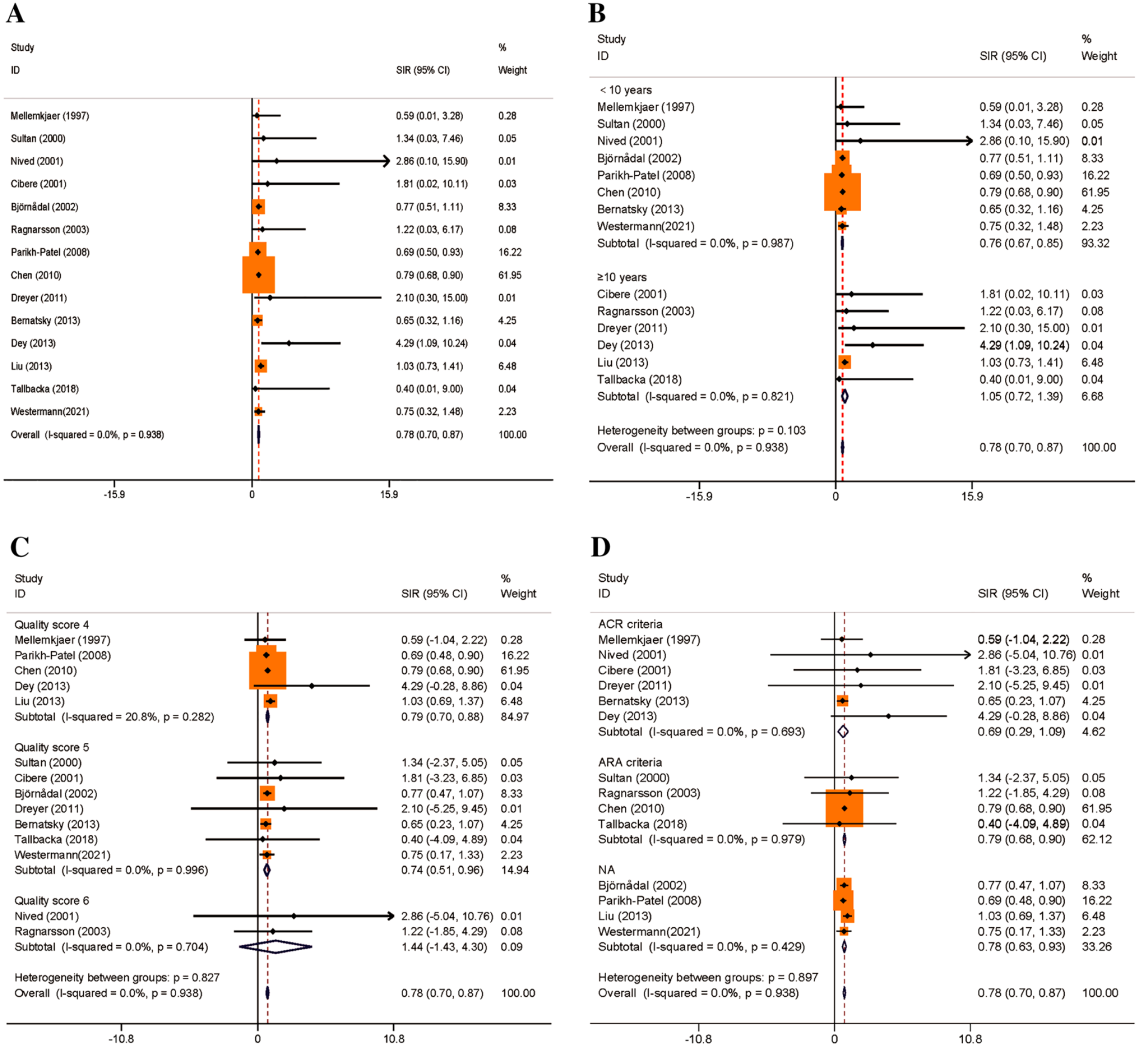
Forest plot of PC risk in patients with SLE and subgroup analysis. **A** overall effect; **B** subgroup analysis of follow-up duration; **C** subgroup analysis of quality score; and **D** subgroup analysis of SLE diagnosis. *SLE* systemic lupus erythematosus, *SIR* standardized incidence rate, *CI* confidence interval, *ACR* American College of Rheumatology, *ARA* American Rheumatism Association, *NA* Not available

In total, 79,316 patients from 14 trials were enrolled in the overall PC risk analysis for SLE. The fixed-effects model of the pooled data indicated no heterogeneity among the studies (I^2^, 0.00%; H^2^, 1; Cochran’s Q test, 6.22; *P* = 0.938). The findings showed that SLE was related to a reduced incidence of PC (SIR, 0.78; 95% CI, 0.70–0.87). Stratified analysis indicated no statistical difference in the efficacy rate among the subgroups of follow-up duration, SLE diagnosis, and quality score (*P* = 0.103, 0.897, and 0.827, respectively). Figure 3 shows a forest map of the SIRs.

#### Sensitivity analysis

Sensitivity analyses were performed to check for robustness by omitting low-quality articles. The results indicated that the studies included in the meta-analysis were stable (Figure S1). Egger’s test (*P* = 0.177) and funnel plots revealed no evidence of publication bias (Figure S2). Meta-regression indicated no variables that might have led to the original heterogeneity (sample size, follow-up time, SLE diagnosis, and quality score).

### Mendelian randomization results

#### Power calculation

Assuming that the SNPs explain 6.45% of the total variation in SLE, the power to detect a causal effect size (SIR = 0.78) for SLE was 100% at a significance level of *P* = 0.05 for our sample size of 79,148 PC cases and 61,106 controls, according to the methods described by Burgess et al (2014). In addition, considering our sample size, there was 100% power to detect a minimal SIR (SIR = 0.4) at a significance level of *P* = 0.05 (Tallbacka et al. 2018). All of the above findings suggest a robust estimate of the causal effect.

#### The causal effect from SLE to PC

**Fig. 4:**
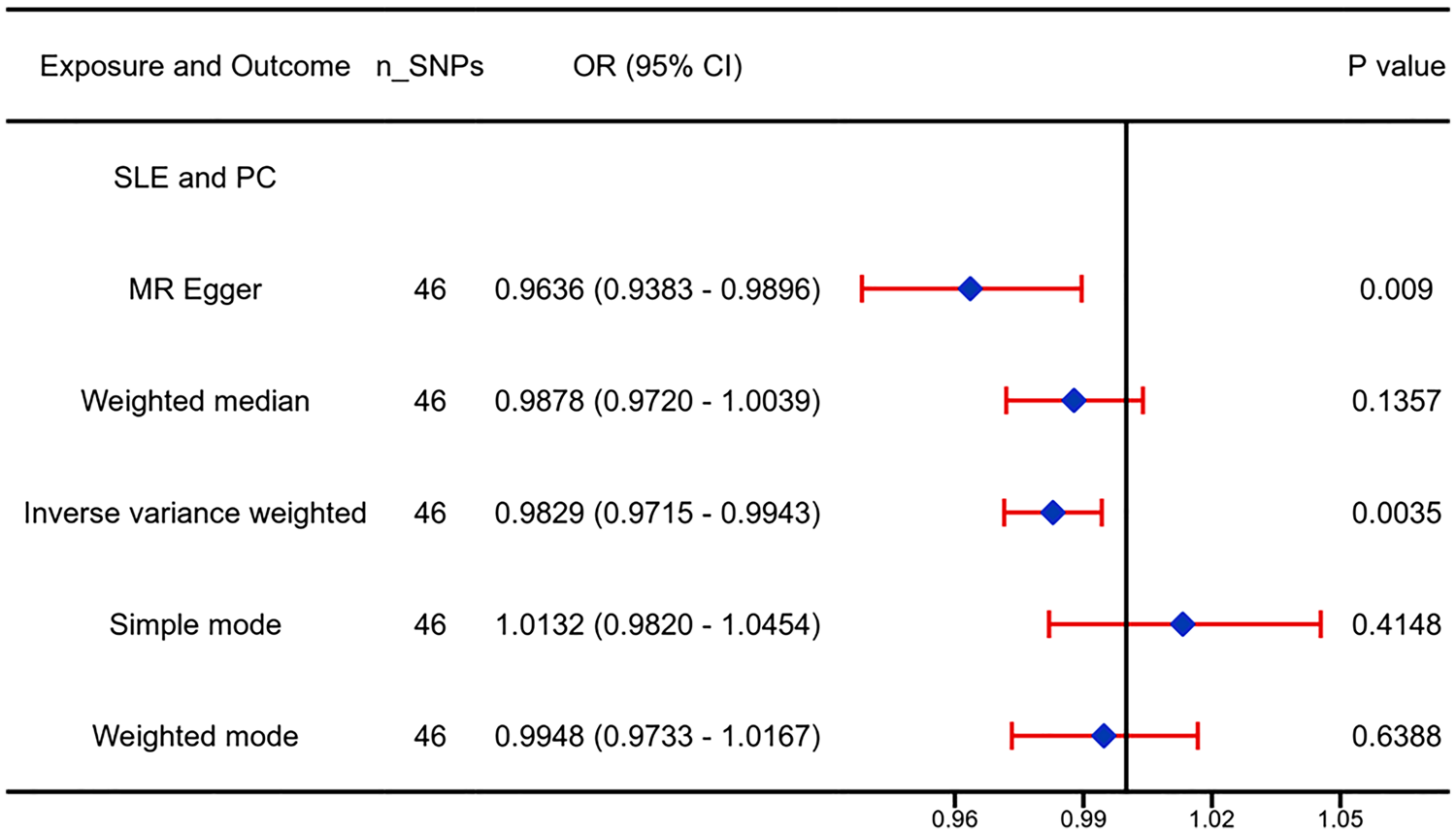
Causal associations between SLE and PC. The number of genetic variants, OR, 95% confidence intervals, *P* values, and *MR* methods of association are shown. *nSNPs* the number of single-nucleotide polymorphisms used as instrumental variables, *OR* combined causal effect, *CI* confidence interval, *P value* *P* value of the causal estimate, *SLE* systemic lupus erythematosus, *PC* prostate cancer

The associations between the 48 selected SNPs and SLE concentrations are shown in Table S2. When genetically predisposed, a one-SD increase in SLE was correlated with a significantly lower PC risk (OR, 0.9829; 95% CI, 0.9715–0.9943; *P* = 0.003) (Fig. 4), which is consistent with the meta-analysis results. The causality estimation of the MR-Egger test (OR, 0.9636; 95% CI, 0.9383–0.9896; *P* = 0.009) was similar in direction and magnitude.

No heterogeneity was detected in the sensitivity analysis (IVW, Q *p*-value = 0.09). The MR-Egger intercept test was used to investigate pleiotropy because the IVs were larger than the three SNPs. MR-Egger regression analysis revealed no directional pleiotropy (*P* = 0.11). Based on SNPs with genome-wide significance and more SNPs of less stringent significance, leave-one-out sensitivity analysis showed that no single SNP drove the overall effect of SLE on PC (Figures S4, S5).

#### Summary of the MR analysis on confounders

**Fig. 5:**
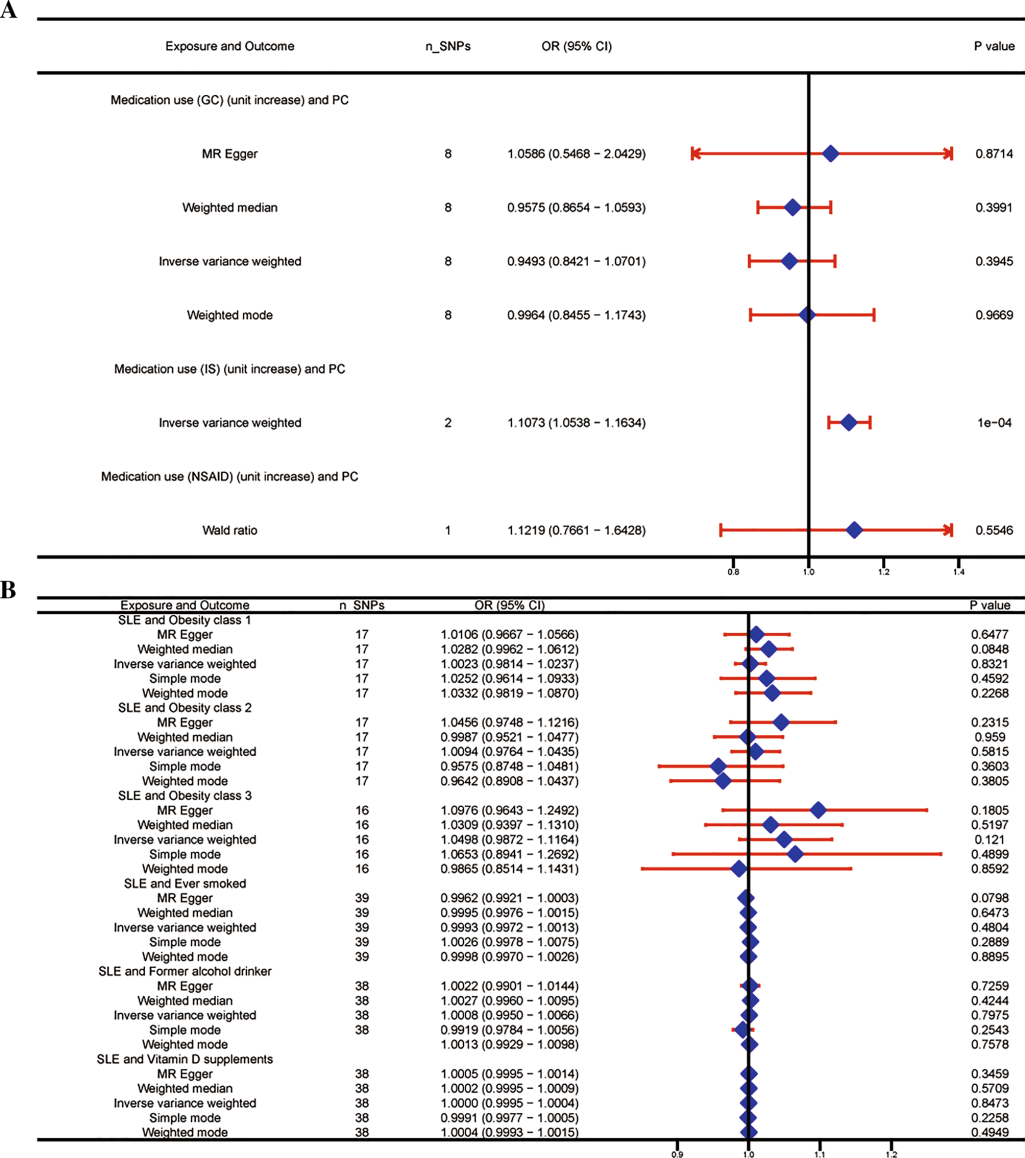
Causal effects of potential confounders on MR analysis. The number of genetic variants, OR, 95% confidence intervals, *P* values, and MR methods of association are shown. *nSNPs* the number of single-nucleotide polymorphism used as instrumental variables, *OR* the combined causal effect, *CI* confidence interval, *P value* *P* value of the causal estimate, *SLE* systemic lupus erythematosus, *PC* prostate cancer, *GCs* glucocorticoids, *ISs* immunosuppressants, *NSAIDs* non-steroidal anti-inflammatory drugs, *NA* not available

Additional MR analyses were performed to determine the potential confounders that affect the causal relationship between genetically predisposed SLE and PC. The results showed that PC was associated with increased IS use (OR, 1.1073; 95% CI, 1.0538–1.1634; *P* < 0.001). However, no causal link was observed between PC and GCs or NSAIDs (GCs: OR, 0.9493; 95% CI, 0.8421–1.0701; *P* = 0.3945; NSAIDs: Not available). The IVW results showed no association between SLE and obesity, smoking, alcohol consumption, or vitamin D supplementation (Fig. 5).

## Discussion

To our knowledge, this is the most comprehensive and extensive assessment of the association between SLE and PC risk. We reviewed 14 cohort studies involving 79,316 patients with SLE. In this study, observational studies and GWAS data showed that a one-SD increase in SLE was causally linked to decreased PC risk. Moreover, our MR results suggest that ISs, but not GCs or NSAIDs, were associated with increased PC risk in patients with SLE. No causal relationship was reported between genetic susceptibility to SLE and potential confounding factors, including obesity, smoking, alcohol consumption, and vitamin D supplementation. This finding enriches our understanding of the potential risk factors for PC in patients with SLE.

As an aberrant inflammatory response is a common clinical feature of SLE and PC, it is easy to infer that patients with SLE may be prone to PC (1). Our meta-analysis (SIR, 0.78; 95% CI 0.70–0.87) and MR analysis (OR, 0.9829; 95% CI, 0.9715–0.9943; *P* = 0.003) further verified the protective effect of the genetic prediction of SLE on PC risk. However, its primary mechanism of action remains unclear. One possible explanation is that SLE and PC may share common pathways and genetic factors. Androgens can mediate cell proliferation and key physiological processes in prostate tissue and are considered essential risk factors for PC (Bu et al. 2016; Pollard et al. 1982; Dobbs et al. 2019). In addition, testosterone levels in men with SLE are lower than those without SLE (4). Low circulating testosterone levels may lower the PC risk in patients with SLE (Watts et al. 2018). Therefore, an altered sex hormone pathway may influence PC development in men with SLE.

In addition, the increased use of ISs in patients with SLE may be a mediating factor. Our MR analyses suggested that the use of ISs (OR, 1.1073; 95% CI, 1.0538–1.1634; *P* < 0.001), but not GCs or NSAIDs, was associated with increased PC risk. Additionally, it is unclear whether the harmful effects of IS on PC are due to inflammation. Since GCs and NSAIDs may suppress inflammation (Watts et al. 2018), ISs may increase PC risk in ways other than simply affecting inflammation. Several studies have shown that calcineurin inhibitors increase the aggressiveness and development of prostate adenocarcinoma tumor cells in vitro and in vivo (Pollard 1997; Hojo et al. 1999). Furthermore, in a rat PC experimental model, cyclosporine enhanced the incidence of metastatic forms via increased production of transforming growth factor, which promotes tumor cell aggressiveness and mobility (Pollard 1997). Consequently, further investigation of the potential causes of PC risk in patients with SLE is required.

Although SLE may reduce PC risk by curtailing the sex hormone pathway (Watts et al. 2018), lower testosterone levels in PC tend to be accompanied by higher aggressive malignancy and a poor prognosis with androgen deprivation therapy (ADT) (Patel 2021; Lane et al. 2008; Huynh et al. 2021). Castration levels of testosterone (Oefelein et al. 2000) below the saturation point (< 0.7 nM) can cause altered differentiation and enhanced metastatic potential in PC cells (Ishiwata et al. 2011; Jennbacken et al. 2010; Wei et al. 2007). Therefore, early attention to PC risk in patients with SLE is of great significance for improving prognosis and mortality in such patients.

Our study has several advantages. First, it covers recently updated research containing data collected over extended follow-up periods. We used various methods to verify the accuracy and reliability of the results. As a result, our study has more robust statistical power than the previous meta-analysis. Second, we stratified the studies according to follow-up duration, SLE diagnosis, and quality score to further control for confounders. Stratified analysis indicated no statistical difference in the efficacy rate among the subgroups of follow-up duration, SLE diagnosis, or quality score (*P* = 0.103, 0.897, and 0.827, respectively). Moreover, our design strictly followed the assumptions of MR (VanderWeele et al. 2014), thus preventing the effects of potential confounding factors and inverse causality and indicating an independent correlation between SLE and PC.

The study population enrolled in the meta-analysis and MR analysis was mainly from Europe, which limits inferences for people of other ancestries. For the meta-analysis, the search strategy was limited to fully published papers written in English, which means certain related articles written in other languages may have been missed. Patients with another malignancy may be less likely to undergo PSA screening, which could lead to an underestimation of the incidence of PC in these patients. Additionally, the diagnosis and mortality of another malignancy and PC could be competitive, which may further impact the observed incidence of PC. Due to the consideration of competing risks in observational studies, we conducted the analysis using an MR study design to investigate the role of SLE in PC. This approach allows for the estimation of the role of SLE without the potential confounding effects of socioeconomic position or other factors, as genetic variants are determined at conception. For the MR study, as there are no data on advanced PC, we could only study the association between SLE and overall PC risk. Despite our finding of a negative correlation between SLE and PC, caution should be exercised when considering their overall effects. Further research is required to confirm this conclusion.

## Conclusion

In conclusion, patients with SLE have a lower risk of developing PC, and the use of ISs may be associated with an increased PC risk. Although SLE may reduce PC risk through the sex hormone pathway, PC in patients with SLE is often highly malignant and has a poor prognosis with ADT. Therefore, early attention to PC risk in patients with SLE is of great significance for improving prognosis and mortality in such patients.

## Supplementary Information

Below is the link to the electronic supplementary material.

Supplementary file1 (DOCX 2362 KB)

## Data Availability

The datasets GWAS for this study can be found in the GWAS Catalog [https://www.ebi.ac.uk/gwas/]. GWAS ID details can be found in methods or Supplementary Material.

## Abbreviations

GWASs: Genome-wide association studies
SLE: Systemic lupus erythematosus
PC: Prostate cancer
GC: Glucocorticoid
IS: Immunosuppressant
NSAIDs: Non-steroidal anti-inflammatory drugs
MR: Mendelian randomization
GIANT: Genetic Investigation of anthropometric traits consortium
MOOSE: Meta-analysis of observational studies in epidemiology
QATSO: Quality assessment tool for systematic reviews of observational studies
RCTs: Randomized controlled trials
SIR: Standardized incidence rate
OR: Odds ratio; HR, hazard ratio
RR: Relative risk; CI, confidence interval
SNP: Single-nucleotide polymorphism
IVs: Instrumental variables
IVW: Inverse variance-weighted
MeSH: Medical subject headings
