# Supplementary material for: Systemic lupus erythematosus and prostate cancer risk: a pool of cohort studies and Mendelian randomization analysis
Source: J Cancer Res Clin Oncol. 2023 May 22;149(12):9517–28. doi: 10.1007/s00432-023-04853-5 (PMC10423167; doi:10.1007/s00432-023-04853-5)

Supplement material

**Electronic search strategy for Pubmed**

**Table S1** Quality score of the enrolled studies in the meta-analysis

**Table S2** The SLE-related genetic variants used for the MR analyses

**Table S3** Association between genetically predisposed systemic lupus erythematosus and potential confounders.

**Figure S1** Sensitivity analysis of Meta-analysis.

**Figure S2** Funnel plot of meta-analysis.

**Figure S3** Scatter plots and forest plot showing the effect of SLE on PC via different methods.

**Figure S4** Leave-one-out analysis of MR analyses

**Figure S5** Funnel plot of of MR analyses

**The code for main analysis which was used in R.**

**PRISMA 2020 checklist of meta-analysis**

**Electronic search strategy for Pubmed**

Search: ((systemic lupus erythematosus) AND (cancer)) AND (((cohort) OR (incidence)) OR (risk))

("lupus erythematosus, systemic"[MeSH Terms] OR ("lupus"[All Fields] AND "erythematosus"[All Fields] AND "systemic"[All Fields]) OR "systemic lupus erythematosus"[All Fields] OR ("systemic"[All Fields] AND "lupus"[All Fields] AND "erythematosus"[All Fields])) AND ("cancer s"[All Fields] OR "cancerated"[All Fields] OR "canceration"[All Fields] OR "cancerization"[All Fields] OR "cancerized"[All Fields] OR "cancerous"[All Fields] OR "neoplasms"[MeSH Terms] OR "neoplasms"[All Fields] OR "cancer"[All Fields] OR "cancers"[All Fields]) AND ("cohort studies"[MeSH Terms] OR ("cohort"[All Fields] AND "studies"[All Fields]) OR "cohort studies"[All Fields] OR "cohort"[All Fields] OR "cohort s"[All Fields] OR "cohorte"[All Fields] OR "cohorts"[All Fields] OR ("epidemiology"[MeSH Subheading] OR "epidemiology"[All Fields] OR "incidence"[All Fields] OR "incidence"[MeSH Terms] OR "incidences"[All Fields] OR "incident"[All Fields] OR "incidents"[All Fields]) OR ("risk"[MeSH Terms] OR "risk"[All Fields]))

Translations

systemic lupus erythematosus: "lupus erythematosus, systemic"[MeSH Terms] OR ("lupus"[All Fields] AND "erythematosus"[All Fields] AND "systemic"[All Fields]) OR "systemic lupus erythematosus"[All Fields] OR ("systemic"[All Fields] AND "lupus"[All Fields] AND "erythematosus"[All Fields])

cancer: "cancer's"[All Fields] OR "cancerated"[All Fields] OR "canceration"[All Fields] OR "cancerization"[All Fields] OR "cancerized"[All Fields] OR "cancerous"[All Fields] OR "neoplasms"[MeSH Terms] OR "neoplasms"[All Fields] OR "cancer"[All Fields] OR "cancers"[All Fields]

cohort: "cohort studies"[MeSH Terms] OR ("cohort"[All Fields] AND "studies"[All Fields]) OR "cohort studies"[All Fields] OR "cohort"[All Fields] OR "cohort's"[All Fields] OR "cohorte"[All Fields] OR "cohorts"[All Fields]

incidence: "epidemiology"[Subheading] OR "epidemiology"[All Fields] OR "incidence"[All Fields] OR "incidence"[MeSH Terms] OR "incidences"[All Fields] OR "incident"[All Fields] OR "incidents"[All Fields]

risk: "risk"[MeSH Terms] OR "risk"[All Fields]

**Supplementary Tables.**

**Table S1** Quality score of the enrolled studies in the meta-analysis

| **Author** | **Country** | **mean or median Study Period(years)** | **Eligibility criteria of selecting participants** | **Appropriate SLE diagnosis** | **Described participants’ characteristics** | **Ascertainment of prostate cancer** | **Adjustments for age and sex** | **Other relevant adjustments** | **Quality score (0-6)** |
| --- | --- | --- | --- | --- | --- | --- | --- | --- | --- |
| Mellemkjaer (1997) |  | 6.8 | Yes | Unclear | Yes | Yes | Yes | No | 4 |
| Sultan (2000) |  | 4.8 | Yes | Yes | Yes | Yes | Yes | No | 5 |
| Nived (2001) |  | 6.5 | Yes | Yes | Yes | Yes | Yes | Yes | 6 |
| Cibere (2001) |  | 12.0 | Yes | Yes | Yes | Yes | Yes | No | 5 |
| Björnådal (2002) |  | 8.8 | Yes | Unclear | Yes | Yes | Yes | Yes | 5 |
| Ragnarsson (2003) |  | 12.8 | Yes | Yes | Yes | Yes | Yes | Yes | 6 |
| Parikh-Patel (2008) |  | 5.1 | Yes | Unclear | Yes | Yes | No | Yes | 4 |
| Chen (2010) |  | 6.1 | Yes | Yes | Yes | Yes | No | No | 4 |
| Dreyer (2011) |  | 13.2 | Yes | Yes | Yes | Yes | Yes | No | 5 |
| Bernatsky (2013) |  | 7.4 | Yes | Yes | Yes | Yes | Yes | No | 5 |
| Dey (2013) |  | 14.7 | Yes | Yes | Yes | Yes | No | No | 4 |
| Liu (2013) |  | 11.3 | Yes | Unclear | Yes | Yes | No | Yes | 4 |
| Tallbacka (2018) |  | 25.7 | Yes | Yes | Yes | Yes | Yes | No | 5 |
| Westermann(2021) |  | 8.1 | Yes | Unclear | Yes | Yes | Yes | Yes | 5 |

**Table S2** The SLE-related genetic variants used for the MR analyses

|  |  | **Exposure：Systemic lupus erythematosus \|\| id:ebi-a-GCST003156** | | | | | **Outcome：Prostate cancer \|\|ebi-a-GCST006085** | | | | | | |
| --- | --- | --- | --- | --- | --- | --- | --- | --- | --- | --- | --- | --- | --- |
| **SNP** | **CHR** | **A1** | **A2** | **Beta** | **SE** | **pval.** | **A1** | **A2** | **Beta** | **SE** | **pval** | **r_pn** | **r2_pn** |
| rs10048743 | 2 | T | G | -0.231112 | 0.0412056 | 2.04E-08 | T | G | 0.0151 | 0.0114 | 0.1859 | 0.046935099 | 0.002202904 |
| rs10200680 | 2 | T | C | -0.248461 | 0.0424835 | 4.96E-09 | T | C | 0.0073 | 0.0118 | 0.5358 | 0.048938432 | 0.00239497 |
| rs1078324 | 5 | A | C | -0.71335 | 0.0781665 | 7.11E-20 | A | C | 0.0173 | 0.0178 | 0.3313 | 0.076299214 | 0.00582157 |
| rs10912578 | 1 | G | A | -0.24686 | 0.0309918 | 1.65E-15 | G | A | 0.0102 | 0.0087 | 0.2421 | 0.066618207 | 0.004437986 |
| rs114012503 | 6 | G | A | 0.41211 | 0.0385365 | 1.09E-26 | G | A | 0.0048 | 0.017 | 0.777 | 0.089359871 | 0.007985187 |
| rs116191291 | 6 | T | C | -0.400478 | 0.032959 | 5.68E-34 | T | C | 0.053 | 0.0173 | 0.00220602 | 0.101473597 | 0.010296891 |
| rs12055602 | 6 | C | T | 0.802002 | 0.0429792 | 1.04E-77 | C | T | -0.0097 | 0.0124 | 0.4362 | 0.155290034 | 0.024114995 |
| rs13136219 | 4 | T | C | -0.174353 | 0.027787 | 3.50E-10 | T | C | -0.01 | 0.0086 | 0.2472 | 0.052500251 | 0.002756276 |
| rs13332649 | 16 | G | A | -0.314711 | 0.0375683 | 5.43E-17 | G | A | -0.0068 | 0.0099 | 0.4889 | 0.070053269 | 0.00490746 |
| rs143123127 | 17 | A | G | 0.470004 | 0.0840342 | 2.23E-08 | A | G | 0.013 | 0.0217 | 0.549201 | 0.046803562 | 0.002190573 |
| rs1464446 | 3 | T | G | -0.328504 | 0.0401497 | 2.79E-16 | T | G | 0.0107 | 0.0104 | 0.3026 | 0.068425805 | 0.004682091 |
| rs150180633 | 6 | T | C | 0.928219 | 0.0689573 | 2.66E-41 | T | C | -0.0561 | 0.0383 | 0.1429 | 0.112347739 | 0.012622015 |
| rs17849501 | 1 | T | C | 0.81093 | 0.0498642 | 1.81E-59 | T | C | -0.0327 | 0.0211 | 0.1214 | 0.135536367 | 0.018370107 |
| rs204899 | 6 | T | C | -0.274437 | 0.0487709 | 1.83E-08 | T | C | 0.0075 | 0.0136 | 0.583201 | 0.047088284 | 0.002217307 |
| rs2299851 | 6 | A | G | -0.415515 | 0.0531931 | 5.65E-15 | A | G | 0.0203 | 0.013 | 0.1199 | 0.065333993 | 0.004268531 |
| rs2431697 | 5 | C | T | -0.223144 | 0.0292964 | 2.60E-14 | C | T | -0.0057 | 0.0087 | 0.5093 | 0.063708906 | 0.004058825 |
| rs2459611 | 2 | T | C | 0.261365 | 0.045245 | 7.62E-09 | T | C | 0.0076 | 0.0139 | 0.585 | 0.048338603 | 0.002336621 |
| rs268124 | 2 | T | C | 0.18633 | 0.0323703 | 8.60E-09 | T | C | 0.0054 | 0.0091 | 0.5507 | 0.048167622 | 0.00232012 |
| rs2736332 | 8 | C | G | 0.277632 | 0.0320694 | 4.83E-18 | C | G | 0.015 | 0.0092 | 0.1034 | 0.072390256 | 0.005240349 |
| rs28752924 | 6 | C | T | -0.248461 | 0.030032 | 1.30E-16 | C | A | -0.0105 | 0.0149 | 0.4813 | 0.069187248 | 0.004786875 |
| rs3128929 | 6 | T | C | 0.198851 | 0.0338103 | 4.07E-09 | T | C | -0.0105 | 0.0094 | 0.264 | 0.049213749 | 0.002421993 |
| rs34703115 | 2 | C | T | -0.616186 | 0.104778 | 4.08E-09 | C | T | 0.0147 | 0.0252 | 0.5586 | 0.049209832 | 0.002421608 |
| rs35000415 | 7 | T | C | 0.587787 | 0.041539 | 1.86E-45 | T | C | -0.0011 | 0.0129 | 0.9322 | 0.118062951 | 0.01393886 |
| rs35251378 | 19 | A | G | -0.235722 | 0.0324266 | 3.61E-13 | A | G | 0.0042 | 0.0091 | 0.6481 | 0.060809335 | 0.003697775 |
| rs353608 | 11 | G | A | 0.18633 | 0.0280198 | 2.93E-11 | G | A | 0.0027 | 0.0083 | 0.741199 | 0.05563557 | 0.003095317 |
| rs3747093 | 22 | A | G | 0.262364 | 0.0345055 | 2.88E-14 | A | G | -0.0041 | 0.01 | 0.6806 | 0.063598611 | 0.004044783 |
| rs41263824 | 6 | A | G | -0.385662 | 0.0371039 | 2.64E-25 | A | G | -0.0017 | 0.0091 | 0.8495 | 0.086863416 | 0.007545253 |
| rs4388254 | 5 | T | C | 0.378436 | 0.0603977 | 3.71E-10 | T | C | -0.037 | 0.0185 | 0.0453304 | 0.052425869 | 0.002748472 |
| rs45527431 | 6 | G | A | 0.587787 | 0.0499459 | 5.68E-32 | G | A | -0.0513 | 0.0146 | 0.000429705 | 0.098296438 | 0.00966219 |
| rs4661543 | 1 | G | T | 0.274437 | 0.0423755 | 9.40E-11 | G | T | 0.0049 | 0.0133 | 0.713899 | 0.05418512 | 0.002936027 |
| rs4916215 | 1 | T | C | 0.223144 | 0.0339693 | 5.07E-11 | T | C | 0.0052 | 0.0092 | 0.573801 | 0.054959236 | 0.003020518 |
| rs501480 | 6 | C | T | 0.198851 | 0.0296764 | 2.07E-11 | C | T | 0.0033 | 0.008 | 0.677599 | 0.056059231 | 0.003142637 |
| rs512681 | 2 | A | C | -0.301105 | 0.0421192 | 8.75E-13 | A | C | -0.0076 | 0.011 | 0.4919 | 0.059802843 | 0.00357638 |
| rs58688157 | 11 | G | A | -0.223144 | 0.0335647 | 2.97E-11 | G | A | -0.0058 | 0.0094 | 0.5378 | 0.055620561 | 0.003093647 |
| rs58721818 | 6 | T | C | 0.65752 | 0.0755941 | 3.38E-18 | T | C | -0.0341 | 0.0252 | 0.1767 | 0.072730623 | 0.005289744 |
| rs597808 | 12 | G | A | -0.162519 | 0.0294736 | 3.51E-08 | G | A | 0.0202 | 0.0082 | 0.0139399 | 0.04614351 | 0.002129223 |
| rs6671847 | 1 | A | G | 0.198851 | 0.0289651 | 6.64E-12 | A | G | -0.0049 | 0.0082 | 0.5486 | 0.057433507 | 0.003298608 |
| rs6679677 | 1 | A | C | 0.336472 | 0.0464854 | 4.55E-13 | A | C | 0.016 | 0.0134 | 0.2351 | 0.060548805 | 0.003666158 |
| rs6889239 | 5 | C | T | 0.277632 | 0.03174 | 2.19E-18 | C | T | -0.0079 | 0.0092 | 0.3902 | 0.073139472 | 0.005349382 |
| rs7097397 | 10 | A | G | -0.18633 | 0.0287118 | 8.60E-11 | A | G | -0.0014 | 0.0085 | 0.8732 | 0.054296525 | 0.002948113 |
| rs73050535 | 12 | T | C | -0.71335 | 0.124134 | 9.11E-09 | T | C | 0.0193 | 0.0332 | 0.5612 | 0.048087432 | 0.002312401 |
| rs73068668 | 19 | A | G | -0.314711 | 0.0574903 | 4.40E-08 | A | G | 0.0475 | 0.016 | 0.002939 | 0.045809985 | 0.002098555 |
| rs7386188 | 8 | G | T | 0.494296 | 0.0865089 | 1.10E-08 | G | T | 0.0046 | 0.022 | 0.8357 | 0.047813405 | 0.002286122 |
| rs7768653 | 6 | T | C | -0.207014 | 0.0296891 | 3.11E-12 | T | C | -0.0079 | 0.0085 | 0.3522 | 0.058331826 | 0.003402602 |
| rs7823055 | 8 | T | G | -0.350657 | 0.0286208 | 1.64E-34 | T | G | -0.0023 | 0.0087 | 0.7899 | 0.102312899 | 0.010467929 |
| rs78517564 | 1 | C | A | -0.328504 | 0.0515759 | 1.90E-10 | C | A | 0.0212 | 0.0128 | 0.098951 | 0.053291391 | 0.002839972 |
| rs9265290 | 6 | C | A | 0.277632 | 0.0384304 | 5.04E-13 | C | A | 0.0042 | 0.0133 | 0.7502 | 0.060432371 | 0.003652072 |
| rs9461633 | 6 | G | A | -0.235722 | 0.0362595 | 7.98E-11 | G | A | -0.0014 | 0.01 | 0.8886 | 0.054391219 | 0.002958405 |
|  |  |  |  |  |  |  |  |  |  |  |  |  |  |

SNP: single nucleotide polymorphism, CHR: chromosome, A1: effect allele, A2: other allele, BETA: beta-coefficient (in standard deviation units), SE: standard error,

**Table S3** Association between genetically predisposed systemic lupus erythematosus and potential confounders.

|  | **ID.exposure** | **ID.outcome** | **Outcome** | **Exposure** | **Method** | **nsnp** | **B** | **SE** | **P** | **Lo CI** | **Up CI** | **OR** | **OR-low CI** | **OR-up CI** |
| --- | --- | --- | --- | --- | --- | --- | --- | --- | --- | --- | --- | --- | --- | --- |
| 1 | ebi-a-GCST003156 | ieu-a-90 | Obesity class 1 | SLE | MR Egger | 17 | 1.06E-02 | 2.27E-02 | 6.48E-01 | -3.39E-02 | 5.51E-02 | 1.01E+00 | 9.67E-01 | 1.06E+00 |
| 2 | ebi-a-GCST003156 | ieu-a-90 | Obesity class 1 | SLE | Weighted median | 17 | 2.78E-02 | 1.61E-02 | 8.48E-02 | -3.81E-03 | 5.94E-02 | 1.03E+00 | 9.96E-01 | 1.06E+00 |
| 3 | ebi-a-GCST003156 | ieu-a-90 | Obesity class 1 | SLE | Inverse variance weighted | 17 | 2.28E-03 | 1.08E-02 | 8.32E-01 | -1.88E-02 | 2.34E-02 | 1.00E+00 | 9.81E-01 | 1.02E+00 |
| 4 | ebi-a-GCST003156 | ieu-a-90 | Obesity class 1 | SLE | Simple mode | 17 | 2.49E-02 | 3.28E-02 | 4.59E-01 | -3.94E-02 | 8.92E-02 | 1.03E+00 | 9.61E-01 | 1.09E+00 |
| 5 | ebi-a-GCST003156 | ieu-a-90 | Obesity class 1 | SLE | Weighted mode | 17 | 3.26E-02 | 2.59E-02 | 2.27E-01 | -1.82E-02 | 8.35E-02 | 1.03E+00 | 9.82E-01 | 1.09E+00 |
| 6 | ebi-a-GCST003156 | ieu-a-91 | Obesity class 2 | SLE | MR Egger | 17 | 4.46E-02 | 3.58E-02 | 2.32E-01 | -2.55E-02 | 1.15E-01 | 1.05E+00 | 9.75E-01 | 1.12E+00 |
| 7 | ebi-a-GCST003156 | ieu-a-91 | Obesity class 2 | SLE | Weighted median | 17 | -1.25E-03 | 2.44E-02 | 9.59E-01 | -4.91E-02 | 4.66E-02 | 9.99E-01 | 9.52E-01 | 1.05E+00 |
| 8 | ebi-a-GCST003156 | ieu-a-91 | Obesity class 2 | SLE | Inverse variance weighted | 17 | 9.35E-03 | 1.70E-02 | 5.82E-01 | -2.39E-02 | 4.26E-02 | 1.01E+00 | 9.76E-01 | 1.04E+00 |
| 9 | ebi-a-GCST003156 | ieu-a-91 | Obesity class 2 | SLE | Simple mode | 17 | -4.34E-02 | 4.61E-02 | 3.60E-01 | -1.34E-01 | 4.69E-02 | 9.58E-01 | 8.75E-01 | 1.05E+00 |
| 10 | ebi-a-GCST003156 | ieu-a-91 | Obesity class 2 | SLE | Weighted mode | 17 | -3.64E-02 | 4.04E-02 | 3.80E-01 | -1.16E-01 | 4.28E-02 | 9.64E-01 | 8.91E-01 | 1.04E+00 |
| 11 | ebi-a-GCST003156 | ieu-a-92 | Obesity class 3 | SLE | MR Egger | 16 | 9.31E-02 | 6.60E-02 | 1.81E-01 | -3.64E-02 | 2.23E-01 | 1.10E+00 | 9.64E-01 | 1.25E+00 |
| 12 | ebi-a-GCST003156 | ieu-a-92 | Obesity class 3 | SLE | Weighted median | 16 | 3.04E-02 | 4.73E-02 | 5.20E-01 | -6.22E-02 | 1.23E-01 | 1.03E+00 | 9.40E-01 | 1.13E+00 |
| 13 | ebi-a-GCST003156 | ieu-a-92 | Obesity class 3 | SLE | Inverse variance weighted | 16 | 4.86E-02 | 3.14E-02 | 1.21E-01 | -1.28E-02 | 1.10E-01 | 1.05E+00 | 9.87E-01 | 1.12E+00 |
| 14 | ebi-a-GCST003156 | ieu-a-92 | Obesity class 3 | SLE | Simple mode | 16 | 6.33E-02 | 8.94E-02 | 4.90E-01 | -1.12E-01 | 2.38E-01 | 1.07E+00 | 8.94E-01 | 1.27E+00 |
| 15 | ebi-a-GCST003156 | ieu-a-92 | Obesity class 3 | SLE | Weighted mode | 16 | -1.36E-02 | 7.52E-02 | 8.59E-01 | -1.61E-01 | 1.34E-01 | 9.87E-01 | 8.51E-01 | 1.14E+00 |
| 16 | ebi-a-GCST003156 | ukb-b-20261 | Ever smoked | SLE | MR Egger | 39 | -3.82E-03 | 2.12E-03 | 7.98E-02 | -7.98E-03 | 3.37E-04 | 9.96E-01 | 9.92E-01 | 1.00E+00 |
| 17 | ebi-a-GCST003156 | ukb-b-20261 | Ever smoked | SLE | Weighted median | 39 | -4.63E-04 | 1.01E-03 | 6.47E-01 | -2.44E-03 | 1.52E-03 | 1.00E+00 | 9.98E-01 | 1.00E+00 |
| 18 | ebi-a-GCST003156 | ukb-b-20261 | Ever smoked | SLE | Inverse variance weighted | 39 | -7.42E-04 | 1.05E-03 | 4.80E-01 | -2.80E-03 | 1.32E-03 | 9.99E-01 | 9.97E-01 | 1.00E+00 |
| 19 | ebi-a-GCST003156 | ukb-b-20261 | Ever smoked | SLE | Simple mode | 39 | 2.63E-03 | 2.45E-03 | 2.89E-01 | -2.17E-03 | 7.43E-03 | 1.00E+00 | 9.98E-01 | 1.01E+00 |
| 20 | ebi-a-GCST003156 | ukb-b-20261 | Ever smoked | SLE | Weighted mode | 39 | -2.03E-04 | 1.45E-03 | 8.90E-01 | -3.05E-03 | 2.64E-03 | 1.00E+00 | 9.97E-01 | 1.00E+00 |
| 21 | ebi-a-GCST003156 | ukb-b-12654 | Former alcohol drinker | SLE | MR Egger | 38 | 2.19E-03 | 6.19E-03 | 7.26E-01 | -9.95E-03 | 1.43E-02 | 1.00E+00 | 9.90E-01 | 1.01E+00 |
| 22 | ebi-a-GCST003156 | ukb-b-12654 | Former alcohol drinker | SLE | Weighted median | 38 | 2.74E-03 | 3.43E-03 | 4.24E-01 | -3.99E-03 | 9.47E-03 | 1.00E+00 | 9.96E-01 | 1.01E+00 |
| 23 | ebi-a-GCST003156 | ukb-b-12654 | Former alcohol drinker | SLE | Inverse variance weighted | 38 | 7.59E-04 | 2.96E-03 | 7.98E-01 | -5.04E-03 | 6.56E-03 | 1.00E+00 | 9.95E-01 | 1.01E+00 |
| 24 | ebi-a-GCST003156 | ukb-b-12654 | Former alcohol drinker | SLE | Simple mode | 38 | -8.11E-03 | 7.00E-03 | 2.54E-01 | -2.18E-02 | 5.61E-03 | 9.92E-01 | 9.78E-01 | 1.01E+00 |
| 25 | ebi-a-GCST003156 | ukb-b-12654 | Former alcohol drinker | SLE | Weighted mode | 38 | 1.34E-03 | 4.31E-03 | 7.58E-01 | -7.11E-03 | 9.78E-03 | 1.00E+00 | 9.93E-01 | 1.01E+00 |
| 26 | ebi-a-GCST003156 | ukb-b-12648 | Vitamin D supplements | SLE | MR Egger | 38 | 4.71E-04 | 4.93E-04 | 3.46E-01 | -4.95E-04 | 1.44E-03 | 1.00E+00 | 1.00E+00 | 1.00E+00 |
| 27 | ebi-a-GCST003156 | ukb-b-12648 | Vitamin D supplements | SLE | Weighted median | 38 | 2.06E-04 | 3.64E-04 | 5.71E-01 | -5.08E-04 | 9.20E-04 | 1.00E+00 | 9.99E-01 | 1.00E+00 |
| 28 | ebi-a-GCST003156 | ukb-b-12648 | Vitamin D supplements | SLE | Inverse variance weighted | 38 | -4.60E-05 | 2.39E-04 | 8.47E-01 | -5.14E-04 | 4.22E-04 | 1.00E+00 | 9.99E-01 | 1.00E+00 |
| 29 | ebi-a-GCST003156 | ukb-b-12648 | Vitamin D supplements | SLE | Simple mode | 38 | -8.85E-04 | 7.19E-04 | 2.26E-01 | -2.29E-03 | 5.23E-04 | 9.99E-01 | 9.98E-01 | 1.00E+00 |
| 30 | ebi-a-GCST003156 | ukb-b-12648 | Vitamin D supplements | SLE | Weighted mode | 38 | 3.89E-04 | 5.65E-04 | 4.95E-01 | -7.17E-04 | 1.50E-03 | 1.00E+00 | 9.99E-01 | 1.00E+00 |
| 31 | G9IYjw | ebi-a-GCST006085 | Medication use (GC) | PC | MR Egger | 8 | 5.69E-02 | 3.37E-01 | 8.71E-01 | -6.04E-01 | 7.17E-01 | 1.06E+00 | 5.47E-01 | 2.05E+00 |
| 32 | G9IYjw | ebi-a-GCST006085 | Medication use (GC) | PC | Weighted median | 8 | -4.35E-02 | 5.16E-02 | 3.99E-01 | -1.45E-01 | 5.76E-02 | 9.57E-01 | 8.65E-01 | 1.06E+00 |
| 33 | G9IYjw | ebi-a-GCST006085 | Medication use (GC) | PC | Inverse variance weighted | 8 | -5.21E-02 | 6.11E-02 | 3.95E-01 | -1.72E-01 | 6.78E-02 | 9.49E-01 | 8.42E-01 | 1.07E+00 |
| 34 | G9IYjw | ebi-a-GCST006085 | Medication use (GC) | PC | Weighted mode | 8 | -3.60E-03 | 8.38E-02 | 9.67E-01 | -1.68E-01 | 1.61E-01 | 9.96E-01 | 8.45E-01 | 1.17E+00 |
| 35 | qB4cjr | ebi-a-GCST006085 | Medication use (IS) | PC | Inverse variance weighted | 2 | 1.02E-01 | 2.52E-02 | 5.37E-05 | 5.24E-02 | 1.51E-01 | 1.11E+00 | 1.05E+00 | 1.16E+00 |
| 36 | CEJibS | ebi-a-GCST006085 | Medication use (NSAID) | PC | Wald ratio | 1 | 1.15E-01 | 1.95E-01 | 5.55E-01 | -2.66E-01 | 4.96E-01 | 1.12E+00 | 7.66E-01 | 1.64E+00 |

SNPs, single-nucleotide polymorphisms; B: beta-coefficient (in standard deviation units); SE: standard error; P: P-value; OR, odds ratio; SLE, Systemic lupus erythematosus; PC, Prostate cancer; GC, glucocorticoids; IS, immunosuppressants; NSAIDs, non-steroidal anti-inflammatory drugs;

**Supplementary Figures**

**Figure S1** Sensitivity analysis of Meta-analysis.


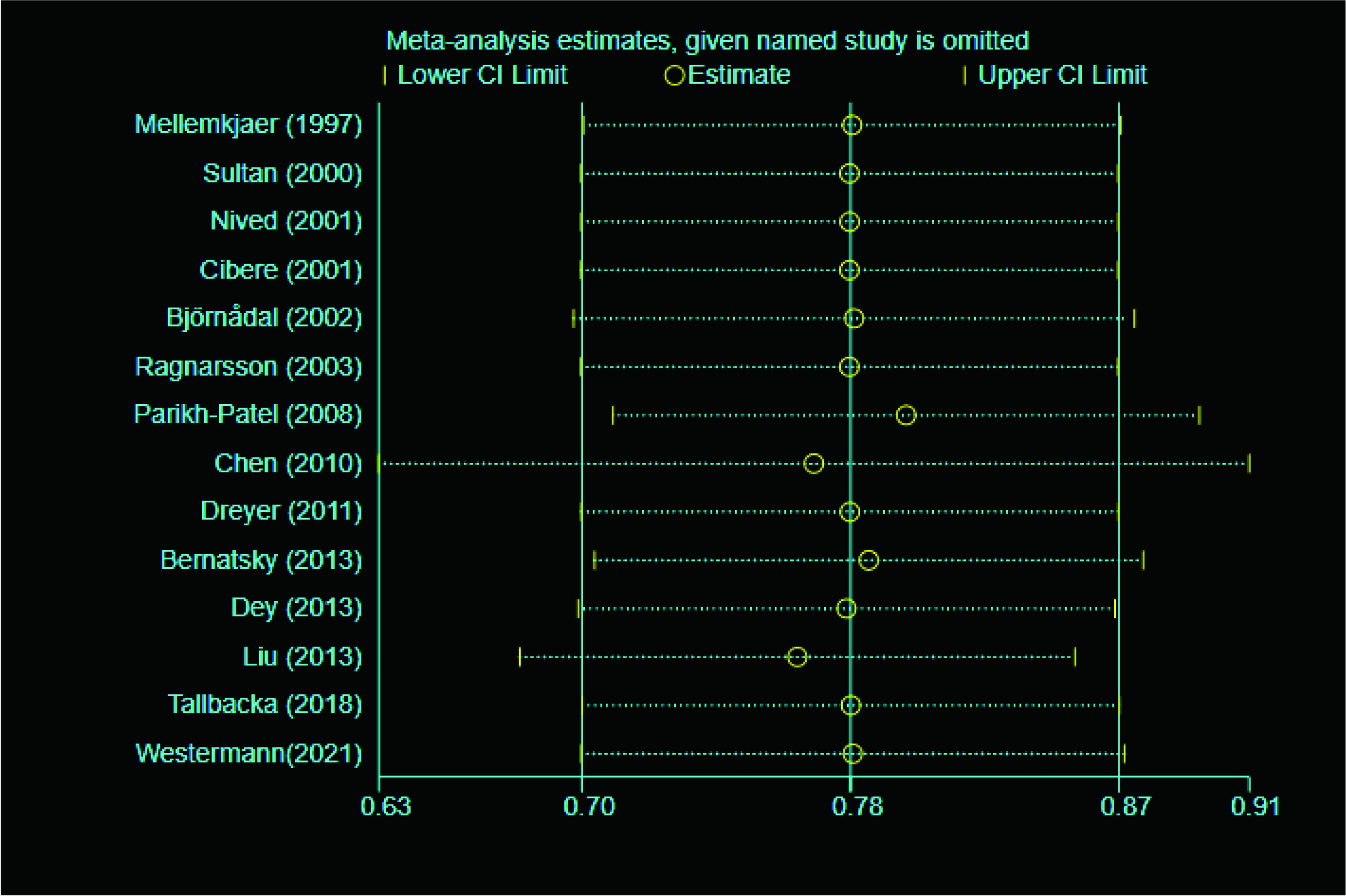


**Figure S2** Funnel plot of meta-analysis.


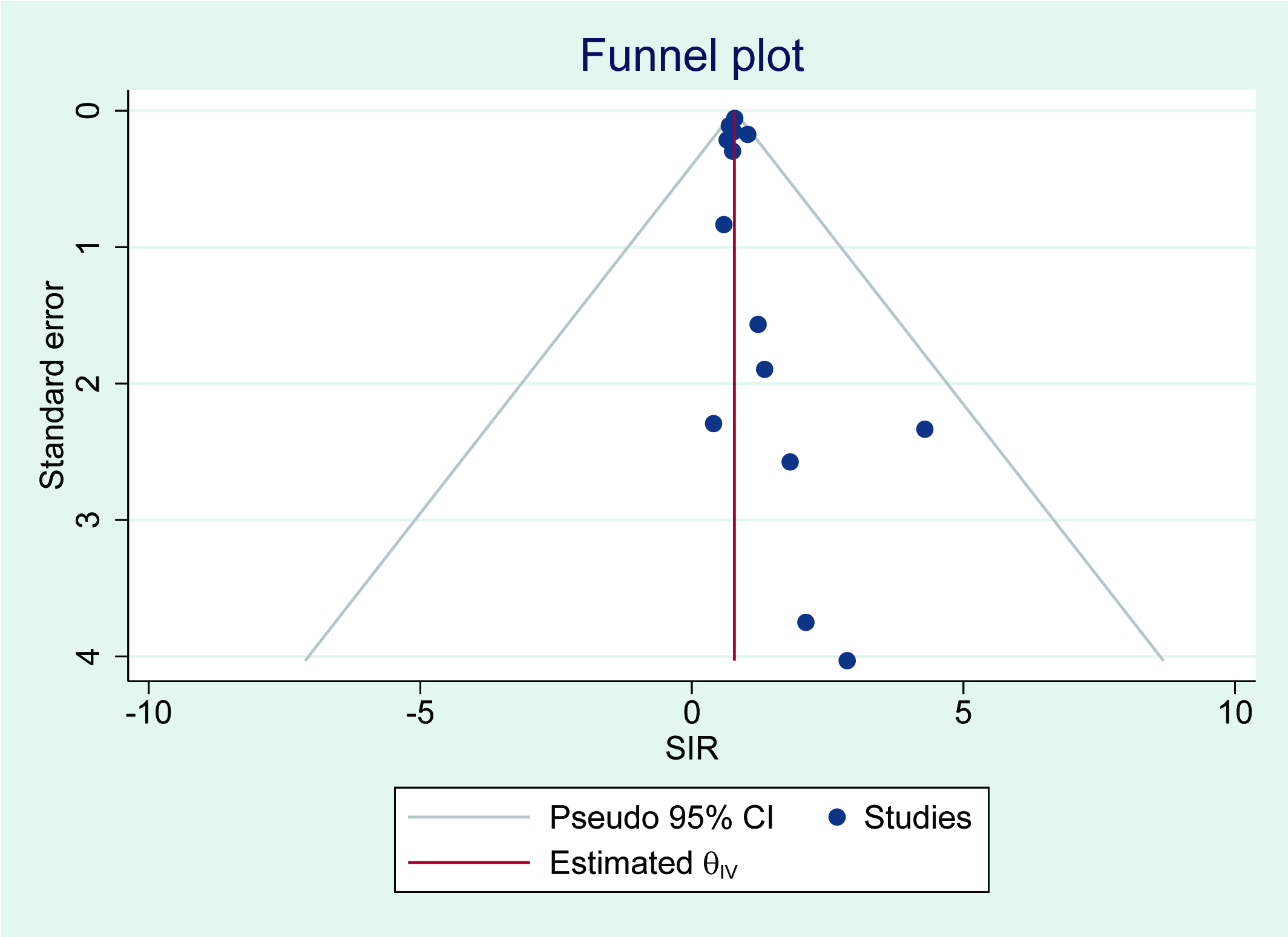


**Figure S3** Scatter plots and forest plot showing the effect of SLE on PC via different methods.


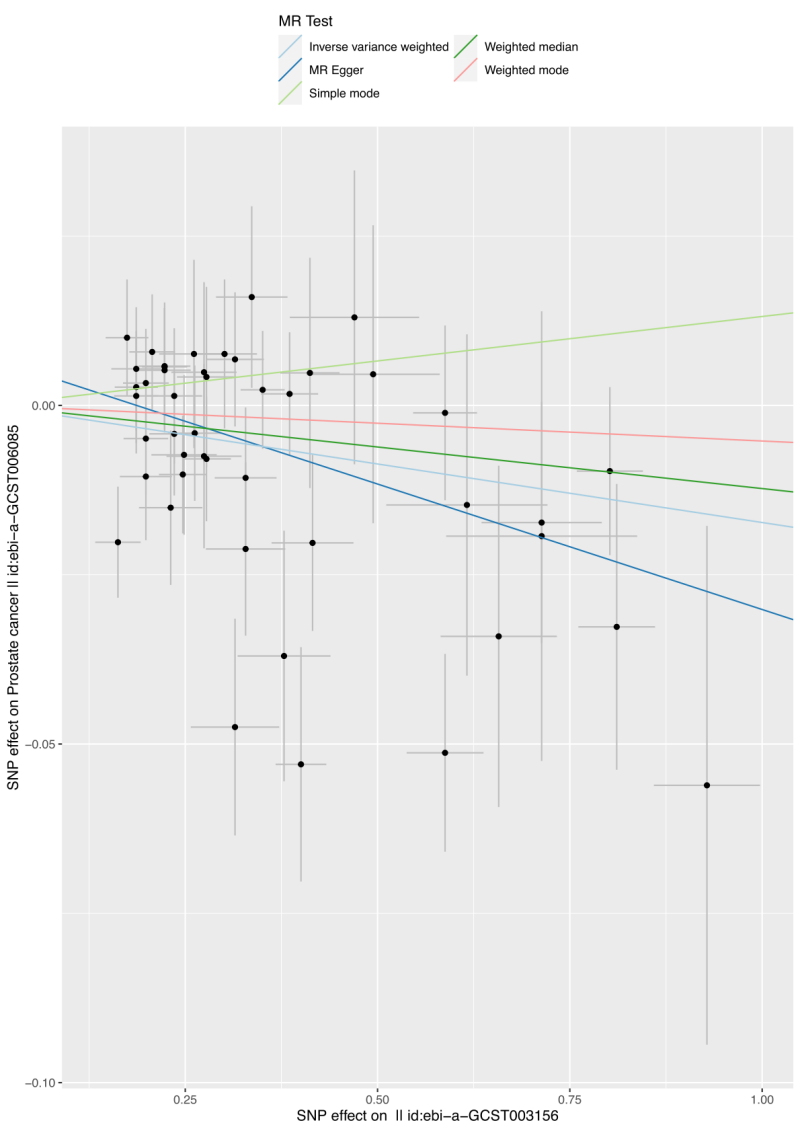


**Figure S4** Leave-one-out analysis of MR analyses


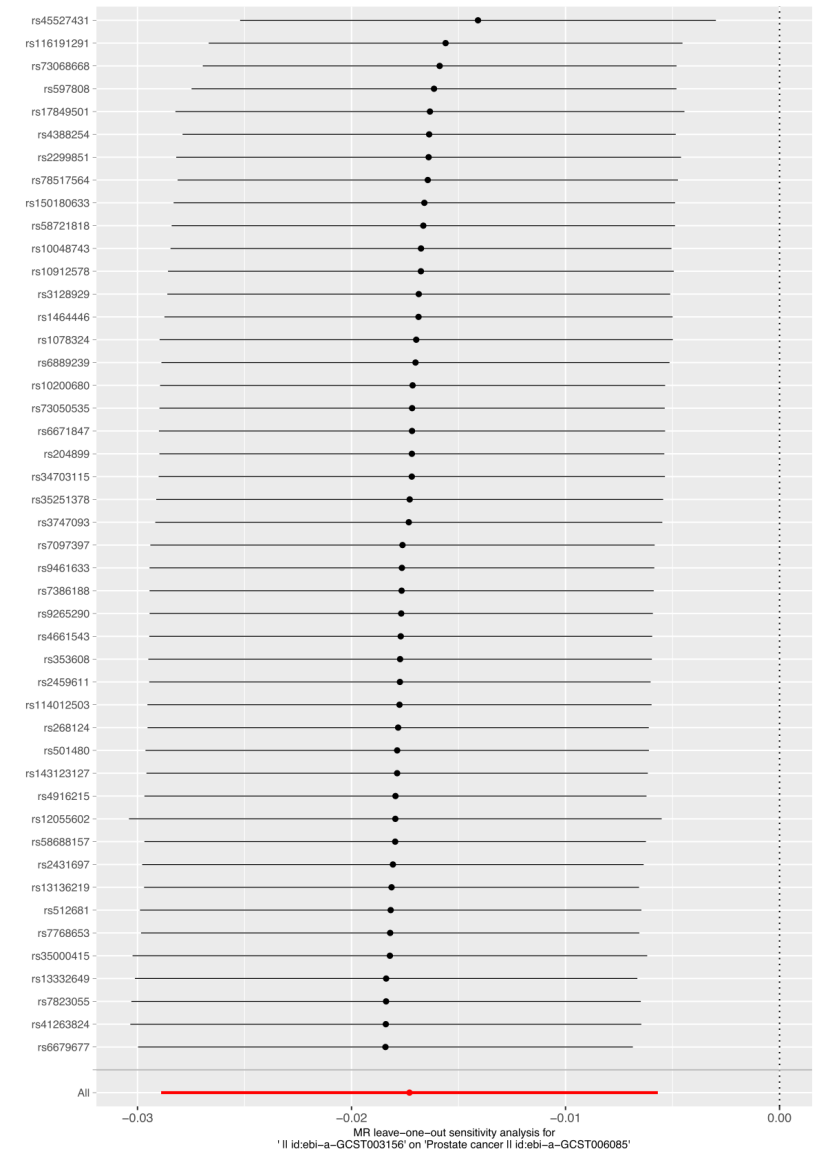


**Figure S5** Funnel plot of of MR analyses


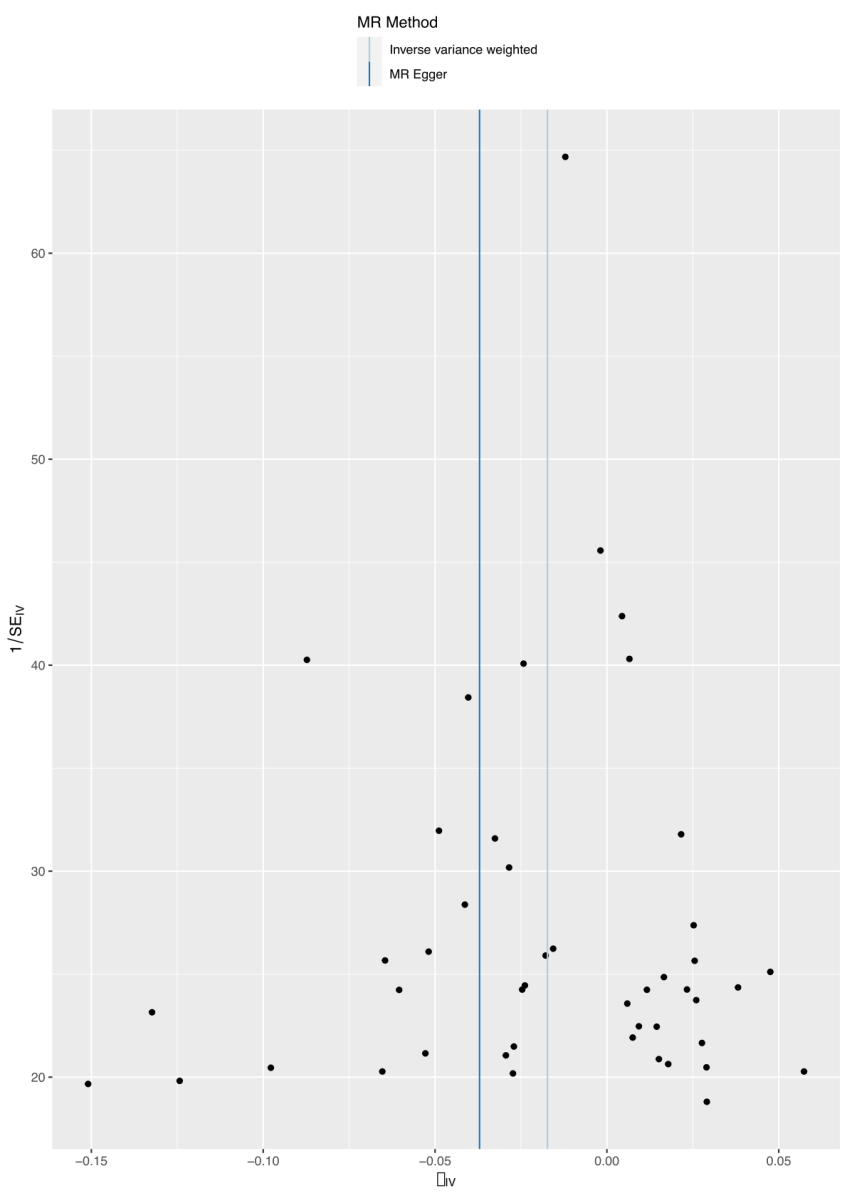


**R code of MR**

#########InstallPackages###################

install.packages("remotes")

library(remotes)

install.packages("devtools")

library(devtools)

install_github("MRCIEU/TwoSampleMR")#choose 3:null#choose 3:null

library(TwoSampleMR)

library(MRInstruments) mrbase

ao <- available_outcomes()

head(ao)

write.csv(ao, "MRBase_GWAS database.csv", row.names=F, quote=F)

library(data.table)

library(reader)

########ReadingExposureData###############

exposure <- extract_instruments(outcomes='ebi-a-GCST003156',clump=TRUE, kb=5000, r2=0.01,p1=5e-8,access_token = NULL)

dim(SLE)

#########GetingR2##############

exposure$r_pn <- get_r_from_pn(exposure$pval.exposure, exposure$samplesize.exposure)

exposure$r2_pn <- exposure$r_pn*exposure$r_pn

write.csv(exposure,file='sle.csv')

#########RemovingConfounderByPhenosccanner去除混杂###########

#Vascular or heart problems diagnosed by doctor: high body mass index, alcohol consumption, smoking, vitamin D supplements and medication use (GC, IS, and NSAIDs)

#The rest SNPs are in the file: Fivelipids_NoConfounder.csv

exposure<-read.csv("Fivelipids_NoConfounder.csv")

########ReadingOutcomeData###########

outcome <-extract_outcome_data(

snps = exposure$SNP,

outcomes ="ebi-a-GCST006085",

proxies = FALSE,

maf_threshold = 0.01,

access_token = NULL)

##########Harmonize#####################

mydata <- harmonise_data(

exposure_dat=exposure,

outcome_dat=outcome,

action= 2

)

##################MR#####################

cmethod <- c("mr_simple_median","mr_weighted_median","mr_egger_regression","mr_ivw_mre")##or"mr_ivw_fe"

res <- mr(mydata)

res

OR<-generate_odds_ratios(res)

#############MRPRESSO####################

if (!require("devtools")) { install.packages("devtools") } else {}

devtools::install_github("rondolab/MR-PRESSO")

library(MRPRESSO)

mr_presso(BetaOutcome ="beta.outcome", BetaExposure = "beta.exposure",

SdOutcome ="se.outcome", SdExposure = "se.exposure",

OUTLIERtest = TRUE,DISTORTIONtest = TRUE, data =mydata,

NbDistribution = 1000, SignifThreshold = 0.05)

##########SensitiveAnalysis#################

het <- mr_heterogeneity(mydata)

het

#mr(mydata,method_list=c('mr_ivw_mre')) #if het IVW Pval<0.05

pleio <- mr_pleiotropy_test(mydata)

pleio

single <- mr_leaveoneout(mydata)

mr_leaveoneout_plot(single)

########visual########

mr_scatter_plot(res,mydata)

res_single <- mr_singlesnp(mydata)

mr_forest_plot(res_single)

mr_funnel_plot(res_single)

write.csv(OR,file='sle-OR.csv')

write.csv(mydata,file='sle-mydata.csv')

**PRISMA 2020 checklist of meta-analysis**


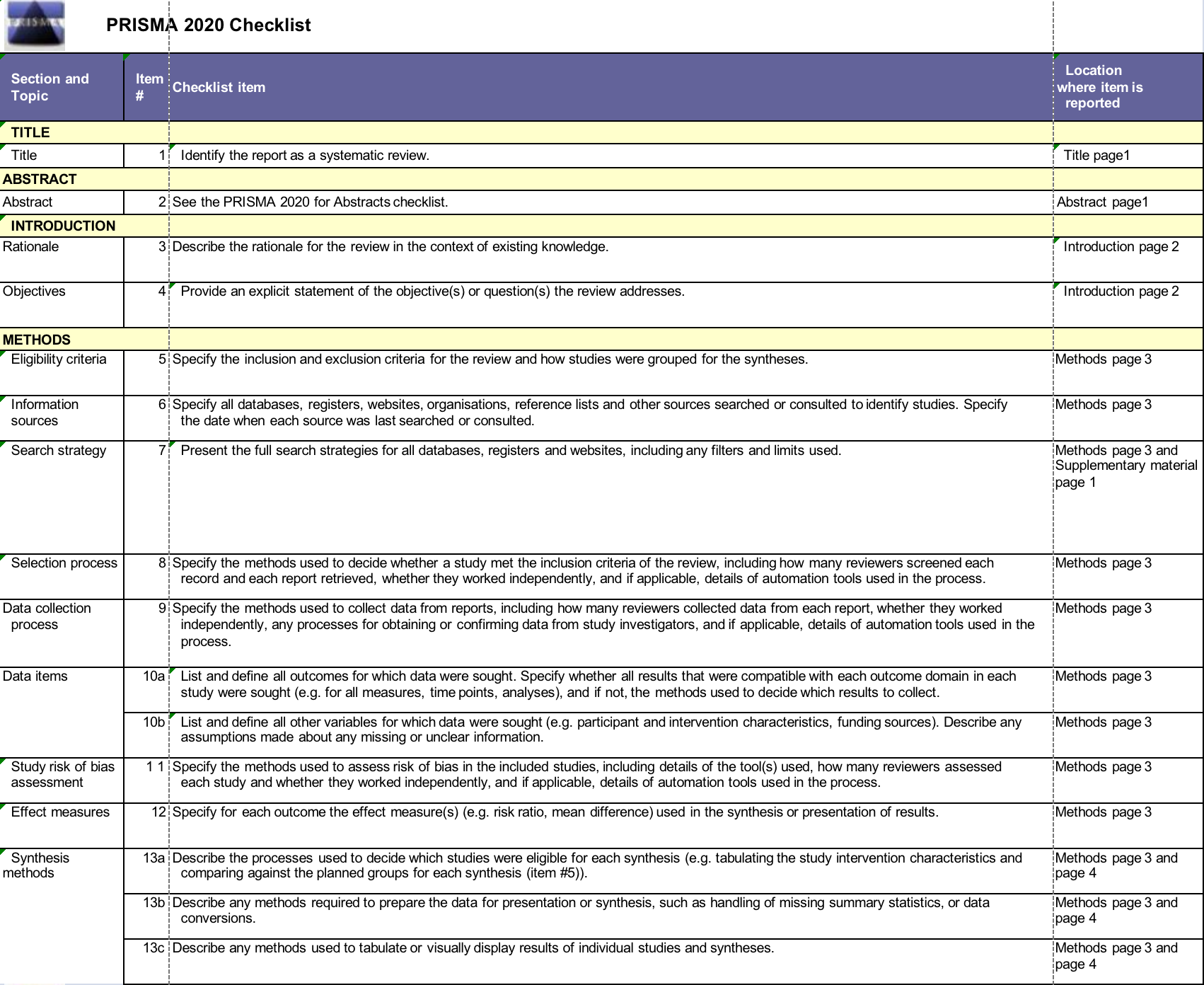


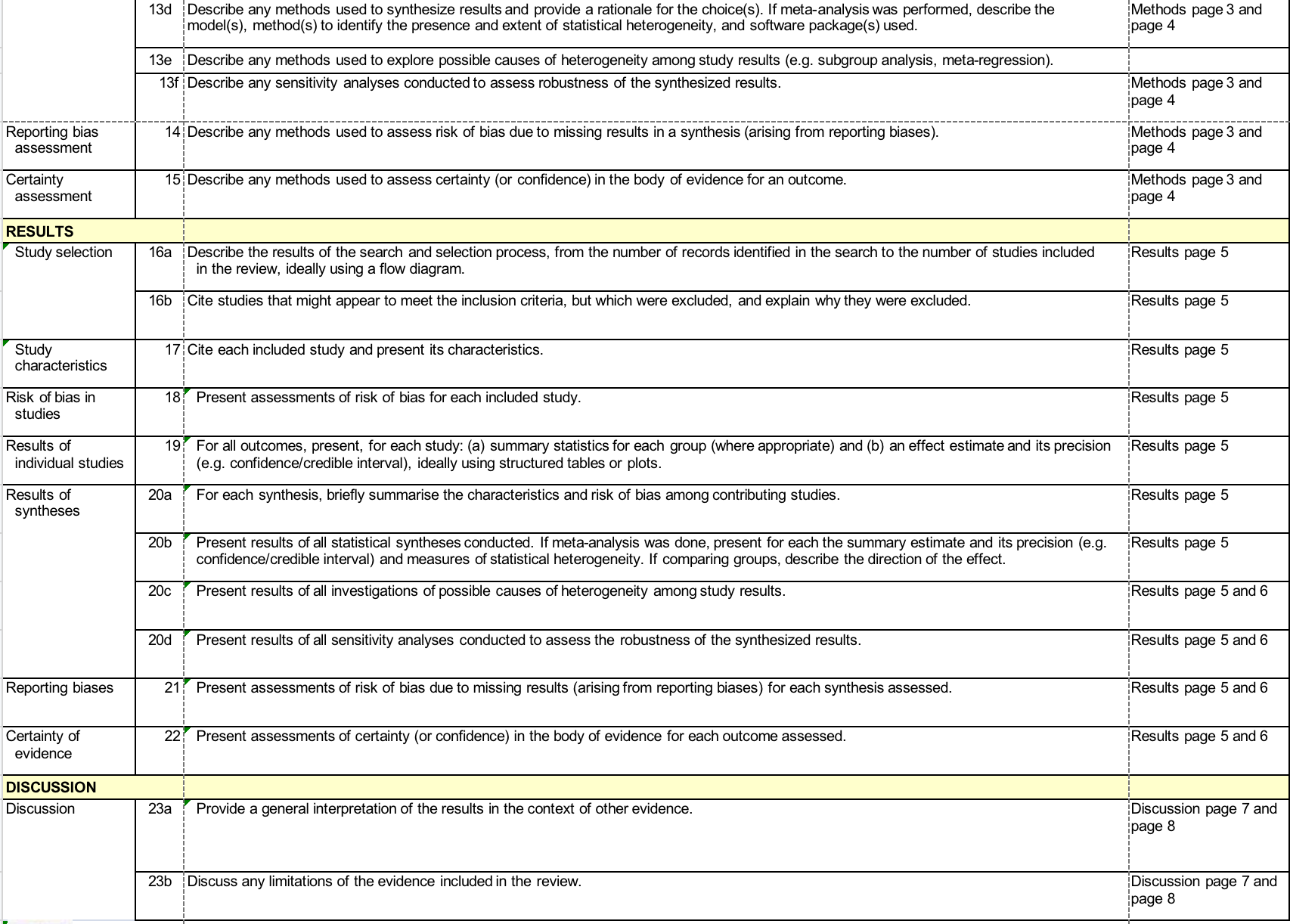

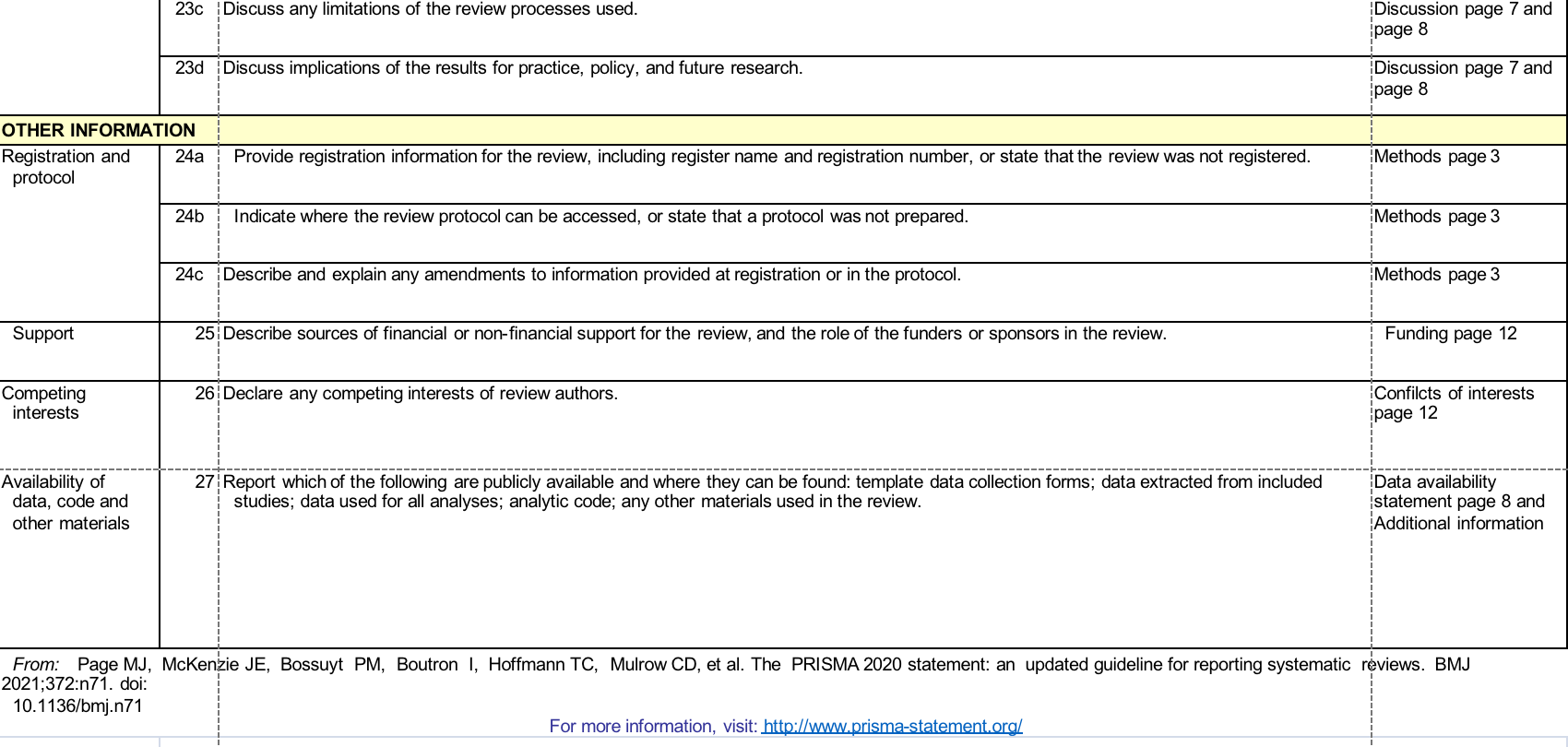

Supplement: Supplementary file 1 — Supplementary file1 (DOCX 2362 KB) [file 432_2023_4853_MOESM1_ESM.docx]
